# Supplementary material for: Complex‐centric proteome profiling by SEC‐SWATH‐MS
Source: Mol Syst Biol. 2019 Jan 14;15(1):e8438. doi: 10.15252/msb.20188438 (PMC6346213; doi:10.15252/msb.20188438)
Supplement: Supplementary file 8 — Dataset EV7 [file MSB-15-e8438-s008.zip › feature_plots_string/Q96KG9.pdf]

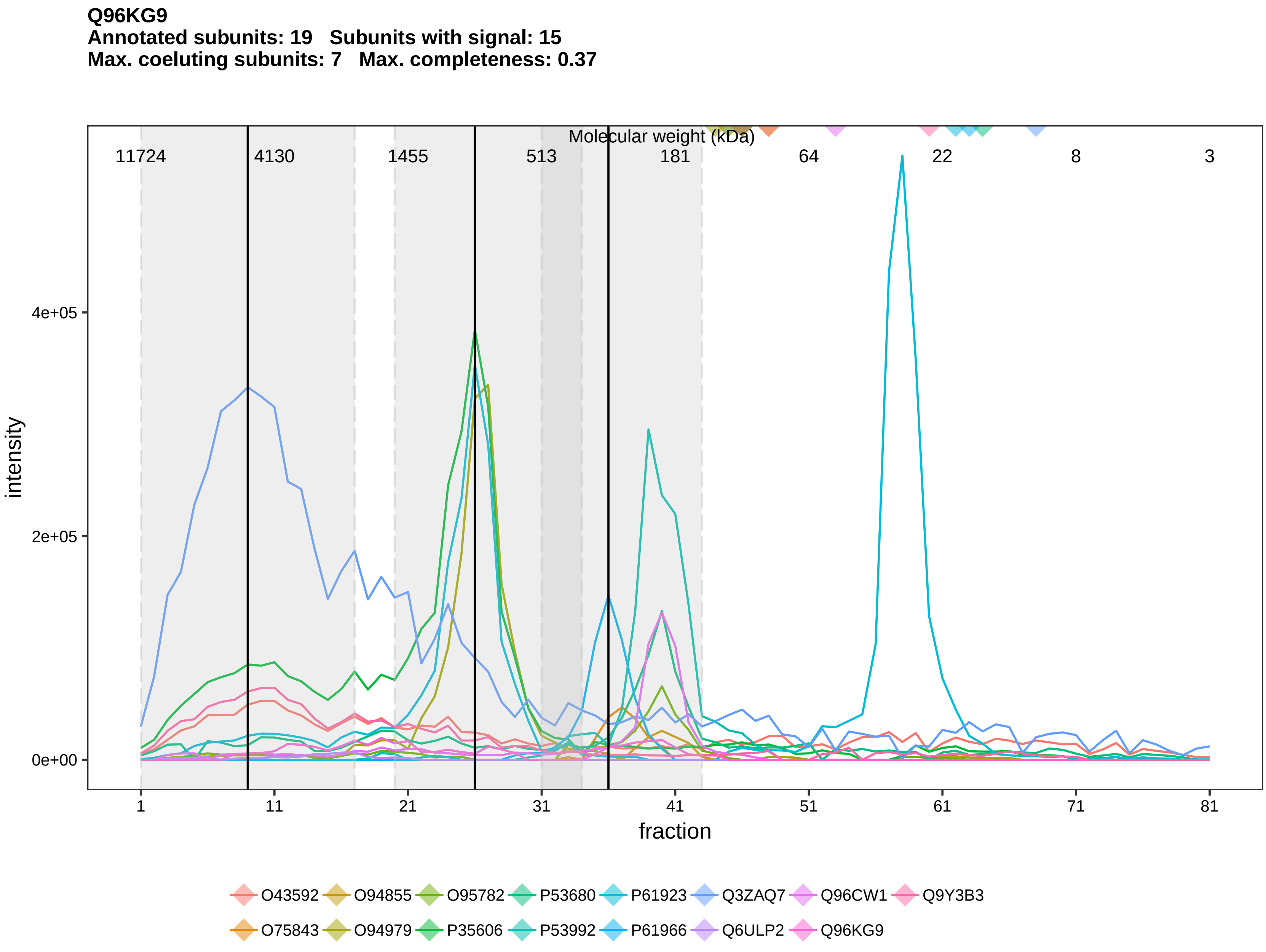
Q96KG9
Annotated subunits: 19 Subunits with signal: 15
Max. coeluting subunits: 7 Max. completeness: 0.37
Molecular weight (kDa)
11724
4130
1455
513
181
64
22
8
3
intensity
4e+05
2e+05
0e+00
1
11
21
31
41
51
61
71
81
fraction
O43592
O94855
O95782
P53680
P61923
Q3ZAQ7
Q96CW1
Q9Y3B3
O75843
O94979
P35606
P53992
P61966
Q6ULP2
Q96KG9
